# Long-chain bases, phosphatidic acid, MAPKs, and reactive oxygen species as nodal signal transducers in stress responses in *Arabidopsis*

**DOI:** 10.3389/fpls.2015.00055

**Published:** 2015-02-13

**Authors:** Mariana Saucedo-García, Marina Gavilanes-Ruíz, Oscar Arce-Cervantes

**Affiliations:** ^1^Instituto de Ciencias Agropecuarias, Universidad Autónoma del Estado de Hidalgo, Tulancingo, Hidalgo, México; ^2^Departamento de Bioquímica, Facultad de Química, Universidad Nacional Autónoma de México, México City, México

**Keywords:** long-chain bases, phosphatidic acid, MAPKs, reactive oxygen species

## Abstract

Due to their sessile condition, plants have developed sensitive, fast, and effective ways to contend with environmental changes. These mechanisms operate as informational wires conforming extensive and intricate networks that are connected in several points. The responses are designed as pathways orchestrated by molecules that are transducers of protein and non-protein nature. Their chemical nature imposes selective features such as specificity, formation rate, and generation site to the informational routes. Enzymes such as mitogen-activated protein kinases and non-protein, smaller molecules, such as long-chain bases, phosphatidic acid, and reactive oxygen species are recurrent transducers in the pleiotropic responses to biotic and abiotic stresses in plants. In this review, we considered these four components as nodal points of converging signaling pathways that start from very diverse stimuli and evoke very different responses. These pleiotropic effects may be explained by the potentiality that every one of these four mediators can be expressed from different sources, cellular location, temporality, or magnitude. Here, we review recent advances in our understanding of the interplay of these four specific signaling components in *Arabidopsis* cells, with an emphasis on drought, cold and pathogen stresses.

## INTRODUCTION

Plants are typically exposed to wide variations of temperature, sunlight, moisture, nutrient availability, and invasive microorganisms from season to season and throughout their entire life cycle on a daily basis. The stressful character of these environmental changes impose a necessary adaptation response by the plant. Due to their inherent lack of motility, plants cannot move to more favorable locations, adapting instead to the prevailing environmental conditions. As a result, they have developed sophisticated and robust mechanisms to detect and react to changes in their surroundings. The ensuing adaptation response encompasses the sensitive detection of environmental insults, the engagement of fast and accurate signal transducers, and the activation and deployment of appropriate effectors. These distinct stages are orchestrated by evolutionarily ancient and remarkably fine-tuned molecular relays. The successful outcome of the entire process depends on the affinity of the receptor for the signaling mediators elicited by a particular insult, as well as the efficient transmission of this signal by specific transducers, such as kinases, phosphatases, transcription factors, genes, and second messenger transducers (McAinsh and Pittman, 2009; Tena et al., 2011). The extensive body of literature on the signaling and transduction pathways in eukaryotic cells had made it clear that many transducers are shared by multiple pathways responding to different stimuli, ultimately becoming informational molecular networks in the course of evolution (Hetherington and Bardwell, 2011; Lumba et al., 2014; Trotta et al., 2014). This review examines the intricate *Arabidopsis* signaling pathway system utilized by plants in response to abscisic acid (ABA) or to messenger molecules elaborated upon exposure to cold or pathogens.

Although an extensive body of literature has documented the signaling pathways involved in the plant responses to drought, cold, or pathogens (see excellent reviews by Mahajan and Tuteja, 2005; Testerink and Munnik, 2005; Neill et al., 2008; Wang and Song, 2008; Hubbard et al., 2010; Krasensky and Jonak, 2012; Lim et al., 2012; Suzuki et al., 2012; Bellin et al., 2013; Meng and Zhang, 2013; Golldack et al., 2014), a particular focus here is to enhance the interrelationship among certain signal transducers identified in these pathways, i.e., long-chain bases (LCBs), phosphatidic acid (PA), reactive oxygen species (ROS), and MAPK (mitogen-activated protein kinase) cascades, which are convergent points.

## RESPONSE TO DROUGHT MEDIATED BY STOMATA CLOSURE

Stomata are key structures involved in the normal physiology of the plant, as they participate in CO_2_, O_2_, and water exchange, as well as in pathogen exposure. Thus, the opening, closure and even size of stomata are highly regulated (Schroeder et al., 2001). Stomata closure is the most important and shortest-term response to a decrease in water availability, since this action prevents the loss of water through the stomatal pore by transpiration (Arve et al., 2011). The main signal that promotes stomata closure is ABA, a first messenger molecule synthesized in leaves and roots in response to water deficiency (Hetherington, 2001). Although ABA is a ubiquitous molecule in the *Plantae* kingdom, it is also found in fungi (Assante et al., 1977) and mammals (Le Page-Degivry et al., 1986). This section focuses on two key signaling lipids, i.e., PA and LCBs, the generation of ROS and the activation of MAPK cascades as it pertains to their role in ABA-mediated stomata closure.

### LCB AND PA AS MEDIATORS OF STOMATA CLOSURE

There are two possible ways in which signal transduction intermediates of lipid nature are generated, either by *de novo* synthesis or by degradation of complex lipids. Therefore, sphingolipids and glycerolipids serve not only as structural building blocks of cell membranes, but they also represent a source of second messengers.

#### Long-chain bases

**FIGURE 1:**
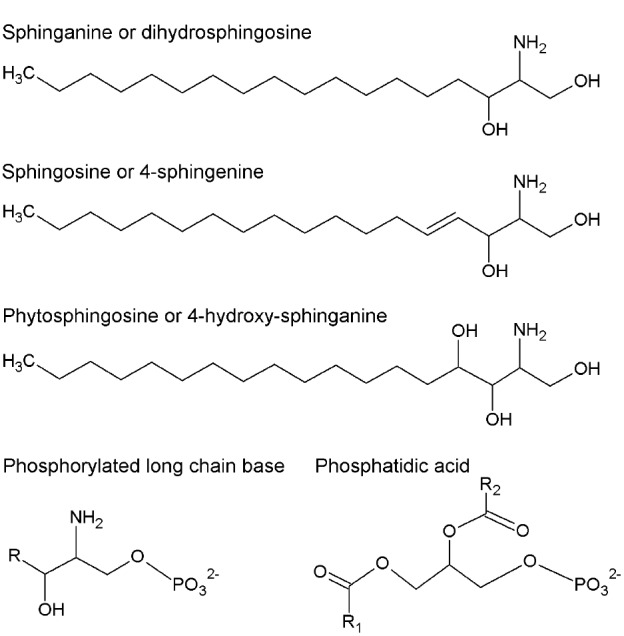
**Chemical structures of long-chain bases (LCB) and phosphatidic acid (PA).** More abundant LCB in plants are shown. They can be in free or phosphorylated form. R, R1, and R2 represent alkyl chains derived from fatty acids.

In contrast to glycerophospholipids, which exhibit a wide distribution throughout phylogeny, sphingolipids are only found in eukaryotic cells. The first step in sphingolipid synthesis requires the condensation of serine and palmitoyl-CoA to yield ketosphinganine, which is subsequently reduced to sphinganine or dihydrosphingosine (DHS), an LCB converted to other modified forms (Figure 1). LCBs are long alkyl chains with an amine group at C2 and at least two hydroxyl groups at C1 and C3. The chain length and the number and position of unsaturations and a third hydroxyl group are very diverse (Markham et al., 2006). LCB esterification with a phosphate group at C1 occurs very often to form phosphorylated LCBs (LCB-Ps) and these can be dephosphorylated by an LCB-P phosphatase or cleaved by an LCB-P lyase, yielding a long-chain aldehyde and ethanolamine phosphate (Saba et al., 1997; Tsegaye et al., 2007; Chen et al., 2008, 2009). LCB-Ps have been defined as signaling components in a wide variety of physiological processes in animal cells, especially in the control of cell proliferation (Olivera and Spiegel, 1993) and programmed cell death (PCD; Cuvillier et al., 1996). In plants, however, during the last 14 years have LCB-Ps come to occupy a higher functional relevance in light of evidence supporting their role as second messengers (Ng et al., 2001), thereby conferring sphingolipids a signaling function in plants together with the structural one in membranes.

The *Arabidopsis* genome includes at least three genes encoding LCB kinases responsible for producing LCB-Ps. Among these, the LCB kinase LCBK1 (At5g51290; Nishiura et al., 2000) utilizes DHS, sphingosine (SPH or 4E-sphingenine), 4,8-sphingadienine and phytosphingosine (PHS or 4D-hydroxy-sphinganine; Figure 1) as substrates in decreasing order of specificity (Imai and Nishiura, 2005). The kinase designated as SPHK1 (At4g21540) preferentially uses SPH and PHS as substrates, reflecting its homology with the human sphingosine kinase 1 (Worrall et al., 2008). Although it was originally assumed that the At4g21540 gene generated only one transcript (Worrall et al., 2008), it is now known that the At4g21540 locus actually contains two distinct genes, *SPHK1* and *SPHK2*, whose products exhibit a characteristic tonoplast subcellular localization. *SPHK1* and *SPHK2* are involved in the ABA response, since disruption of either gene is associated with diminished ABA-induced stomata closure compared to wild-type plants (Worrall et al., 2008; Guo et al., 2012). In contrast, *LCBK1* transcripts slightly increase under low humidity conditions or by exposure to exogenous ABA, suggesting that this isoform is not involved in ABA signaling (Imai and Nishiura, 2005).

Although the first evidence of the role of LCBs in the signaling of plants was reported for stomata closure, the precise identity of the LCB-P involved remains unclear. Consistent with low SPH concentration and low gene expression levels of *SPH Δ4-DESATURASE*–which product catalyzes the conversion of saturated LCB to the unsaturated form (Michaelson et al., 2009)—it appears that SPH-P may not be the bioactive molecule involved in stomata closure as previously reported by Ng et al. (2001) in *Commelina communis* and by Coursol et al. (2003) in *Arabidopsis thaliana*. To clarify this discrepancy, a mutant with a T-DNA insertion in the *Δ4-DESATURASE* gene was generated in *Arabidopsis* and exposed to ABA. No difference in the observed response between this mutant and wild-type plants was found (Michaelson et al., 2009). Thus, SPH-P can be excluded as the signaling mediator in stomata closure, leaving the question open as to which LCB species is responsible for this function in *Arabidopsis*. In particular, PHS-P has been suggested as a possible candidate (Coursol et al., 2005), which could account for the relative abundance of PHS in *Arabidopsis* (Markham et al., 2006). However, the involvement of other LCB cannot be ruled out given their extensive diversity in plants (Lynch et al., 2009) and the increasing reports on differential effects of individual LCB forms in response to various stressors (see below).

#### Phosphatidic acid

**FIGURE 2:**
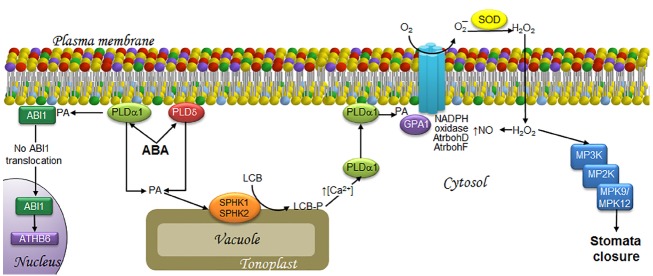
**LCBs, PA, MAPKs, and ROS in the signaling pathway involved in ABA-induced stomata closure in response to drought.** ABA is synthesized as a signal upon water deficit and then is perceived by the guard cells that form the stoma. This is followed by the transduction events illustrated as follows. At the end, the MAPK cascade activation seems responsible of activating a slow anion channel that triggers membrane depolarization that is necessary to close the stoma. The signaling of ABA induces the synthesis of PA by PLDα1 and PLDδ. A fraction of PA in the plasma membrane recruits ABI1, an ABA signaling inhibitor, thereby preventing its translocation to the nucleus, and thus, activation of the transcription factor ATH6. Another fraction of PA is translocated to the tonoplast, where it activates SPHK1/2 leading to LCB-P production. The surge in LCB-Ps causes an increase in cytosolic calcium levels, which favors PLDα1 translocation from the cytosol to the plasma membrane, where it is further activated, enhancing PA levels. PA in turns binds to GPA1, leading to activation of the AtrbohD/F NADPH oxidases. The ensuing production of H_2_O_2_ results in increased levels of NO and the activation of a MAPK cascade that includes MPK9 and MPK12, which play a key role in stomata closure. ABA, abscisic acid; ABI1, ABA-insensitive protein phosphatase 2C; AtrbohD and AtrbohF, NADPH oxidase isoforms; ATHB6, homeodomain protein; Ca^2+^, calcium ion; GPA1, G protein alpha-subunit; H_2_O_2_, hydrogen peroxide; LCB, free long-chain base; LCB-P, phosphorylated form of long-chain base; MPK, mitogen-activated protein kinase; MP2K, mitogen-activated protein kinase kinase; MP3K, mitogen-activated protein kinase kinase kinase; NADPH oxidase, nicotinamide adenine dinucleotide phosphate-oxidase; NO, nitric oxide; O_2_, molecular oxygen; O_2_^–^, superoxide anion; PA, phosphatidic acid; PLDα1 and PLDδ, α1 and δ isoforms of phospholipase D; SOD, superoxide dismutase; SPHK1/2, sphingosine kinases 1/2.

Phosphatidic acid has been implicated as a signaling molecule in different stress responses in plants (Ryu and Wang, 1996, 1998; Young et al., 1996; Lee et al., 1997; Frank et al., 2000; Munnik et al., 2000; van der Luit et al., 2000; Welti et al., 2002). PA can be synthesized via acylation reactions but in this review, we are focused in the PA generated by two alternative biosynthetic routes. One of them is the hydrolysis of structural glycerophospholipids that may occur via phospholipase D (PLD) action, which cleaves phosphatidylcholine and phosphatidylethanolamine to generate PA and a polar group. The other route involves the action of phospholipase C (PLC), which hydrolyzes phosphoinositides to form diacylglycerol (DAG; Athenstaedt and Daum, 1999). The latter can be used as a substrate for the cognate kinase (DGK) and converted into PA, which is found in low amounts in plant plasma membranes but is one of the most extensively characterized signaling lipids known (Welti et al., 2002). Jacob et al. (1999) demonstrated that PA derived from PLD action, particularly the PLDα1 and PLDδ isoforms (Uraji et al., 2012), promotes stomata closure and inhibits their opening upon ABA treatment. The role of PA in stomata closure has been studied at length. In conjunction with other bound molecules, PA is a positive regulator of the ABA signaling pathway, which leads to stomata closure and/or inhibition of their opening. A clear case is the binding of PA generated by PLDα1 to the ABI1 protein phosphatase, whose activity down-regulates the ABA response. The physical interaction of PA promotes the inhibition of the protein phosphatase activity, thus rendering PA as a positive regulator of the ABA-mediated signaling pathway (Zhang et al., 2004). Exposure of *Arabidopsis* cells to ABA increases PA levels, causing ABI1 to localize to the plasma membrane. However, ABI1 is found in the nuclear compartment in the case of *pldα* null mutants, possibly activating the transcriptional factor ATHB6, which is a down-regulator of ABA signaling (Zhang et al., 2004). These findings suggest that the tethering action of ABI1 by PA prevents ATHB6 activation (Figure 2).

#### LCB- and PA-concerted actions in the signaling associated with stomata closure

Independent studies revealing the involvement of the two lipid molecules PA and LCB-P in ABA-mediated stomata closure point to a possible association that deserves further investigation. While PA binds to SPHK1 and SPHK2 to promote their kinase activity (Guo et al., 2011), the addition of exogenous PA also increases LCB-P levels. These results indicate that ABA-mediated SPHK activation is downstream of PA. In addition, it was demonstrated that the generation of both lipids has a reciprocal feedback loop, since PA addition to the mutant lines *pldα1* or *sphk1-1* or *sphk2-1* (which are defective in the expression of *PLDα1*, *SPKH1* or *SPKH2*, respectively) promotes stomata closure, as opposed to the effect of PHS-P in the *pldα1* mutants, thus concluding that LCB-P is upstream of PLDα1 (Guo et al., 2012). It has been suggested that the attendant increase in LCB-P levels during ABA signaling promotes translocation of PLDα1 from the cytosol to the plasma membrane through a Ca^2+^ surge (McAinsh et al., 1996), which raises PA levels. Thus, this view would support the existence of a positive feedback loop between LCB-Ps and PA (see Guo and Wang, 2012, for more details; Figure 2).

An important signaling mediator related to both lipid molecules in the ABA-mediated pathway is the Gα subunit (GPA1). G proteins or GTP-binding proteins are signaling elements that are also involved in stomata closure. These heterotrimeric proteins are composed of α, β, and γ subunits (Wang et al., 2001). The α subunit is dissociated from the βγ dimer when a receptor, which is coupled to the trimeric protein, receives the signal and catalyzes a GDP–GTP exchange reaction upon dissociation (Bischoff et al., 1999). Any of the two species, α, βγ, or both, may interact with downstream elements to amplify a given signal. LCB-P accumulation is required to promote the GPA1 activity during ABA signaling, since *gpa1* mutants are unable to close stomata upon SPH or DHS treatment (Coursol et al., 2003). In addition, PA generated by PLDα1 interacts with GPA1 to mediate stomata closure in *Arabidopsis* (Mishra et al., 2006; Figure 2).

### SIGNALING ROLE OF ROS IN STOMATA CLOSURE

Both PLDα1 and PA have been implicated in ABA-mediated signaling since they can upregulate the activity of NADPH oxidase, which is also known as respiratory burst oxidase homolog (Rboh) due to its similarity to the NADPH oxidase subunit gp91^phox^. NADPH oxidase is a multisubunit complex that catalyzes the formation of superoxide ion (O_2_^–^) at the plasma membrane (Sang et al., 2001). Depletion of *PLDα1* decreases both PA levels and production of O_2_^–^ by NADPH oxidase in *Arabidopsis* (Sang et al., 2001). Both RbohD and RhboF isoforms contain PA binding domains which, when mutated, compromise ABA-induced ROS generation and stomata closure (Zhang et al., 2009). This indicates that a PA/RbohD and PA/RhboF interaction is essential for producing a burst of ROS needed for stomata closure. Elsewhere, activation of GPA1 is required for ROS generation (Zhang et al., 2011), since ROS elevation upon ABA treatment is not detectable in *gpa-1* mutants. Taken together with the results described in the paragraph above, these findings suggest that PA and GPA1 promote ROS generation by RbohD and RhboF, and that this set of events is downstream of LCB-P accumulation (Figure 2).

In this same signaling pathway, H_2_O_2_ synthesis mediated by the NADPH oxidase isoforms AtrbohD and AtrbohF (Kwak et al., 2003; Bright et al., 2006) is required for downstream signaling mediated by nitric oxide (NO) accumulation (Desikan et al., 2002; Neill et al., 2002; Figure 2), a gas molecule involved in abiotic and biotic stress responses.

### MAPKs AS SIGNAL TRANSDUCERS IN STOMATA CLOSURE

The MAPKs have been described as possible participants in stomata closure (Jammes et al., 2009). These are proteins that catalyze the transfer of monophosphate groups to target proteins, and are therefore included in the superfamily of protein kinases, one of the largest groups known. The *A. thaliana* genome contains more than 1,000 genes encoding protein kinases (Arabidopsis Genome Initiative, 2000).

Mitogen-activated protein kinase cascades are organized at three functional levels. The first element of the cascade is a MAPK kinase kinase (MP3K or MEKK), whose activation is triggered by phosphorylation of its catalytic site. This activated form phosphorylates in turn a MAPK kinase (MP2K or MKK or MEK), which finally activates a MAPK (MPK; Schaeffer and Weber, 1999). MPK activation requires the phosphorylation of the Thr and Tyr residues within their T-E/D-Y motifs (Ichimura et al., 2002), which are located in the VII and VIII subdomains of the catalytic site (Bögre et al., 2000). MPKs also contain a CD domain, which functions as a binding site for MP2Ks, phosphatases and substrate proteins (Tanoue et al., 2000). MPKs are classified into four major groups, A, B, C, and D. The first three contain the T-E-Y sequence while group D harbors the T-D-Y motif. Groups A and B exhibit highly conserved CD domains in their C-terminal region, except group C, where it is modified. In contrast, MPKs from group D lack the CD domain altogether (Ichimura et al., 2002).

Functional complexity of the MAPK cascades in plants is partially explained by the large number of genes encoding these proteins. The *Arabidopsis* genome contains genes for more than 60 MP3Ks, 10 MP2Ks, and at least 20 MPKs (Arabidopsis Genome Initiative, 2000). Notwithstanding their smaller number, not all MPKs have been studied. In fact, the function of MPK9 and MPK12 was reported in 2009 for the first time (Jammes et al., 2009). These MPKs are involved in ABA-mediated stomata closure and their genes are highly expressed in guard cells. Both of these MPKs exhibit functional redundancy, since only the double-mutant *mpk9/mpk12* (but not the single-mutants) showed inhibition of ABA-induced stomata closure, thereby leading to significant water loss due to transpiration. Exogenous addition of H_2_O_2_ to the double-mutant diminished stomata closure as observed in the wild-type plants (Jammes et al., 2009), suggesting that MPK9 and MPK12 are downstream of H_2_O_2_ in ABA signaling (Figure 2). The fact that MPK9 and MPK12 are regarded as belonging to different groups (D and B, respectively) implies a difference in their primary structure despite sharing the same function. It should be interesting to explore the basis for this discrepancy.

## STOMATA CLOSURE AND PLANT IMMUNITY

The phyllosphere is not an innocuous, inert environment. It is instead inhabited by a vast and diverse population of microorganisms, from the extremely pathogenic to some that are actually beneficial to their hosts. Despite the constant interaction between plants and microorganisms, plants hardly develop any disease as a result. In the course of evolution, plants have dynamically implemented modifications or adaptations of their defense mechanisms according to the increased virulence potential of pathogens.

Although a large number of bacteria may thrive and proliferate on plant surfaces, some are able of penetrating the plant tissues through the cuticle layer in order to express their pathogenic potential. Consequently, bacteria search for any existing cuticle openings to gain access to the plant tissues. Since stomata are located in the foliar and stem surfaces in all plants, they constitute the main and more effective entry point for bacteria (Getz et al., 1983; Melotto et al., 2006). This is not to say, however, that stomata represent a permanently open and uncontrolled gate for all kinds of pathogens. In *Arabidopsis*, for example, it has been demonstrated that stomata are rapidly closed upon contact with intact microorganisms such as *Pseudomonas syringae* pv. *tomato* (*Pst*) DC3000. This is also the case upon the addition of pathogenic molecules such as the flagellin-derived peptide flg22 or lipopolysaccharide (LPS), both representatives of the so-called microbe-associated molecular patterns (MAMPs; Melotto et al., 2006). Although fungi have the ability to penetrate the cuticle layer, it has been demonstrated that chitosan—a substance with antifungal activity found in fungi cell walls—induces stomata closure as well (Khokon et al., 2010a). These results have broken the paradigm that stomata have only a passive role during pathogen penetration. Instead, their active role is now considered an important component of the innate immunity response that has evolved in plants.

Stomata closure induced by pathogen elicitors is associated with the control of pathogen entry and hence, with the immune response. However, it has been shown that some pathogens suppress stomata closure to cause disease. Some examples are coronatine (COR), an effector produced by *Pseudomonas syringae* (Melotto et al., 2006), fusicoccin, a toxin produced by *Fusicoccum amygdali* (Emi et al., 2001) and a factor from *Xanthomonas campestris* pv *campestris (Xcc*; Gudesblat et al., 2009).

The role of stomata in plant immunity was not known until the last years. However, it is now clear that stomata represent a key element in the infection process and, therefore, plants have evolved selective and sophisticated mechanisms of pathogen rejection at this level. Likewise, some pathogens have evolved the capacity to respond to stomata closure. This serves to illustrate the force of a selective pressure throughout the evolution of the host-pathogen interaction, which in the case of successful pathogens is manifested as an increase in virulence that manages to overcome this first line of immunity in the plant host.

### LCBs AND ABA AS TRANSDUCERS IN STOMATA CLOSURE TO PREVENT PATHOGEN ENTRY

**FIGURE 3:**
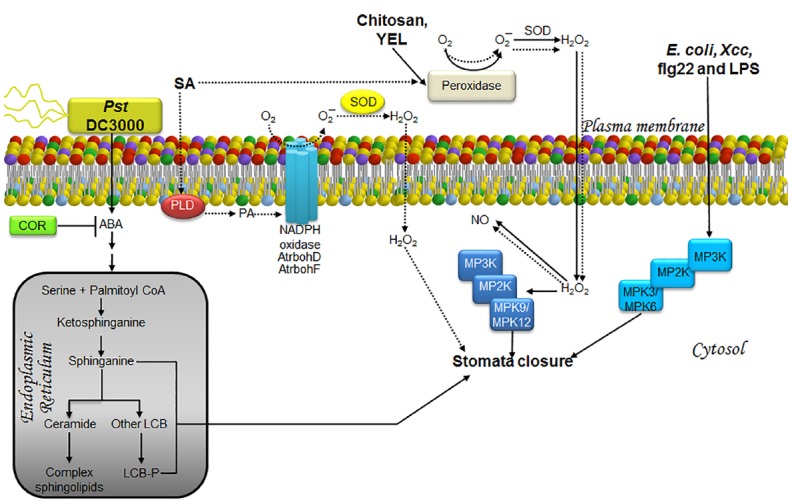
**LCBs, PA, MAPKs, and ROS in the signaling pathway involved in stomata closure as a strategy to prevent pathogen entry during an immunity response.** The schematic shows the main pathway involved in the response to the bacterial strains *Pst* DC3000, *E. coli* and *Xcc*, or to treatment with elicitors such as chitosan, YEL, flg22, LPS (solid lines) and SA, a phytohormone involved in defense responses (dashed lines). In the case of innate immunity, pathogen signals are recognized by receptors surface-located and then information is transmitted as illustrated. Once the information reaches stomata, which is barely understood, they are closed by mechanisms that regulate ion and water fluxes that produce loss of guard cell turgor and then the closing of the central pore. The defense strategy is to close stomata in order to prevent new pathogen entry. The less characterized pathways are those engaged by exposure to *E. coli*, *Xcc*, flg22, and LPS, as well as those comprised in the response to the virulent pathogen *Pst* DC3000. The former have been shown to involve MPK3/6 in stomata closure, whereas the response to *Pst* DC3000 has been shown to require ABA, promoting rapid and transient increases in LCBs within the same time frame as stomata closure (3 h). In the case of chitosan and YEL, stomata closure requires H_2_O_2_ produced by a peroxidase within the apoplast, which then diffuses into the cell causing an increase in NO levels and activation of MPK9/12. Enhanced production of H_2_O_2_ induced by SA is not only mediated by the apoplast peroxidase, but also the catalytic activity of the AtrbohD/F NADPH oxidases. This effect is possibly mediated through the action of PA, whose cellular levels have been observed to increase during SA-induced stomatal closure. No evidence of MAP kinase activation has been documented in this pathway. ABA, abscisic acid; AtrbohD and AtrbohF, NADPH oxidase isoforms; COR, coronatine; *E. coli, Escherichia coli*; flg22, flagellin-derived peptide; H_2_O_2_, hydrogen peroxide; LCB, free long-chain base; LCB-P, phosphorylated form of long-chain base; LPS, lipopolisaccharides; MPK, mitogen-activated protein kinase; MP2K, mitogen-activated protein kinase kinase; MP3K, mitogen-activated protein kinase kinase kinase; NADPH oxidase, nicotinamide adenine dinucleotide phosphate-oxidase; NO, nitric oxide; O_2_, molecular oxygen; O_2_^–^, superoxide anion; PA, phosphatidic acid; PLD, phospholipase D; *Pst*, *Pseudomonas syringae* pv *tomato*; SA, salicylic acid; SOD, superoxide dismutase; YEL, yeast elicitor; *Xcc*, *Xanthomonas campestris *pv *campestris*.

Although evidence for a direct correlation between LCBs and stomata closure as a means to limit pathogen entry has not been found, there are some observations in support of this hypothesis. Thus, Melotto et al. (2006) demonstrated a transient increase in stomata closure when epidermal peels from *Arabidopsis* are exposed to the virulent strain *Pst* DC3000. This effect was reversed after 3 h with an increase in stomata aperture even exceeding that of a positive control, possibly as a result of COR secretion. In addition, Peer et al. (2010) reported a twofold increase of PHS in *Arabidopsis* leaves during the first hour after infiltration with *Pst* DC3000, which subsequently declined to baseline levels over the next 5 h. This suggested that the increase in LCB levels promotes stomata closure, possibly as a defense mechanism (Figure 3).

Abscisic acid mediated signaling is involved in stomata closure caused by *Pst* DC3000 infection, since it is totally abated in the *aba3-1* line, which is an ABA deficient mutant (Melotto et al., 2006). This finding suggests that *Pst* DC3000 induced an increase in ABA levels, leading to a rise in endogenous LCB, thereby promoting stomata closure (Figure 3). Although the involvement of PA is possible, its potential contribution has not been evaluated.

### ROLE OF MAPK IN STOMATA CLOSURE FOR PREVENTING PATHOGEN ACCESS

It appears that chitosan- and yeast elicitor (YEL)-induced stomata closure requires activation of MPK9/MPK12 since this response is abrogated in *mpk9-mpk12* double mutants (Salam et al., 2012, 2013; Figure 3).

Although stomata closure in response to abiotic and biotic stimuli comprises several common elements, there are unique transducers involved in the biotic response. Thus, Gudesblat et al. (2009) demonstrated that MPK3 is required for stomata closure induced by *Escherichia coli, Xcc* and LPS, albeit this MPK is not activated by ABA stimulation. A guard cell-specific antisense *mpk3* mutant is less sensitive to the stomata closure response elicited by these bacteria or LPS and not altered by the presence of ABA, indicating that the role of MPK3 is specific for promoting stomata closure only as an immunity-related effect (Gudesblat et al., 2009). In addition, it was lately demonstrated that although MPK3 is involved in immunity-related stomata closure, genetic ablation of *MPK3* or *MPK6* blocks stomata closure induced by flg22, but not ABA (Montillet et al., 2013; Figure 3). Although MPK4, a well known MPK, has a rapid and transient activation in response to flg22 (Suarez-Rodriguez et al., 2007), this is unable of regulating stomata closure in response to pathogen stress since it has been shown that plants that express MPK4 in a constitutively active form (MPK4DE) exhibit similar stomata aperture than the wild-type plants exposed to flg22 (Berriri et al., 2012).

### ROS GENERATION, A NECESSARY LINK TO STOMATA CLOSURE FOR PREVENTING PATHOGEN INGRESS?

Activation of MPK9 and MPK12 is not involved in ROS generation induced by chitosan since this function remains unaltered in the *mpk9-mpk12* double mutant. However, MPK12 activity increases when H_2_O_2_ is added exogenously (Salam et al., 2012), thereby suggesting that MPK9 and MPK12 activation are located downstream from ROS generation, as in the case of ABA-mediated stomata closure (Salam et al., 2012; Figure 3). All these remarkable similarities suggest that chitosan might increase endogenous levels of ABA to promote stomata closure. However, Issak et al. (2013) reported that chitosan-induced stomata closure is not affected in mutants lacking ABA2, a small alcohol dehydrogenase that is essential for ABA synthesis. This finding indicates that, despite sharing common elements, ABA- or chitosan-induced stomata closure is mediated by two different pathways.

Thus, the question arises as to what makes both signaling pathways different. Once again, a possible answer may lie in the source of ROS generation. In the case of *Arabidopsis*, chitosan-induced stomata closure requires ROS produced by a salicylhydroxamic acid (SHAM)-sensitive peroxidase (Khokon et al., 2010a), and not by the NADPH oxidase isoforms AtrbohD and AtrbohF, as it occurs in the ABA-induced stomata closure (Bright et al., 2006). However, the participation of the NADPH oxidase in ROS production has been reported in *Pisum sativum* in response to chitosan (Srivastava et al., 2009).

The source of ROS production leading to the stomata closure response linked to immunity—i.e., the response triggered by chitosan, YEL, or salicylic acid (SA)—seems to be very similar. In the case of SA, a phytohormone that plays an important role during plant defense against a broad spectrum of pathogens, stomata closure is inhibited upon treatment with catalase (CAT) and superoxide dismutase (SOD; Khokon et al., 2011). Since CAT and SOD are not plasma membrane permeable, the superoxide ion is presumably produced in the extracellular space by the action of a peroxidase, since its activity is inhibited by SHAM treatment (Khokon et al., 2011). The superoxide ion could undergo dismutation thereafter due to SOD activity. Likewise, YEL- or chitosan-induced stomata closure is inhibited by CAT or SHAM treatment, indicating that ROS generation occurs via an extracellular peroxidase (Khokon et al., 2010a,b). Furthermore, SHAM-pretreatment suppresses NO production during the stomata closure response triggered by these three stimuli, indicating that ROS is located upstream of NO generation (Khokon et al., 2010a,b, 2011; Figure 3).

Despite the evidence indicating that a peroxidase is responsible for ROS generation, other possible sources cannot be ruled out. Two of the main sites of ROS formation in plants are the plasma membrane-associated NADPH oxidases and the extracellular peroxidases (Torres and Dangl, 2005). Kalachova et al. (2013) found that SA-induced stomata closure requires the NADPH isoform RbohD, since ablation of its encoding gene only reduced 10% the stomata opening as compared to the untreated mutant, while wild-type plants SA-treated showed a decrease higher than 200% in the opening. Although this evidence is contrary to the report from Khokon et al. (2011), Kalachova et al. (2013) found that the *rbohDrbohF* double mutant failed to exhibit total inhibition on stomata closure in response to SA, pointing to the possible existence of alternate sources of ROS, such as the SHAM-sensitive peroxidase (Kalachova et al., 2013; Figure 3). In this case, the ROS production is essential to stomata closure since the pretreatment with DPI blocked SA-induced stomatal movement in wild-type plants.

Together with the oxidative burst, PA is another key component in the SA-induced stomata closure response. This is illustrated by the addition of the PLD inhibitor 1-butanol, which suppresses both PA accumulation and SA-induced stomata closure. Simultaneous treatment with 1-butanol and H_2_O_2_ causes SA-induced stomata closure, indicating that PA accumulation is upstream of ROS generation (Kalachova et al., 2013), as in the case of ABA-induced stomata closure (Zhang et al., 2009; Figure 3).

Reactive oxygen species production seems to do not have the same function in flg22-induced stomata closure. This last hypothesis was suggested by Berriri et al. (2012) findings, since they observed that ROS production induced by flg22 was strongly reduced in constitutively active MPK4, and as we mentioned above, this mutant did not show differences in comparison with wild type plants on stomatal aperture in response to flg22.

### LCBs, MAPKs, AND ROS AS TRANSDUCERS IN IMMUNITY RESPONSES MEDIATED BY STOMATA CLOSURE

Two important biological tools, Fumonisin B1 (FB1) and *Pst*, have been used in order to dissect the transducing pathways involved in immunity responses.

Fumonisin B1 is a mycotoxin that has been extensively studied due to its structural resemblance to LCBs (Abbas et al., 1994; Merrill et al., 2001). This toxin may be considered a biotic stress factor because it is synthesized by the fungus *Fusarium verticillioides*, a phytopathogen of the *graminea* species that induces a variety of defense-related responses, such as the production of hypersensitive reaction (HR)-like lesions, callose development, phytoalexin accumulation, defense gene expression (Stone et al., 2000), and PCD (Asai et al., 2000; Saucedo-García et al., 2011a).

*Pseudomonas syringae* pv. *tomato* is a gram-negative bacterium widely used in the study of plant-pathogen interactions. *Pst* is also a convenient working model since it can use both tomato plants and *Arabidopsis* as suitable hosts. Because it mainly affects the aerial parts of the plant, such as fruits and leaves in several species, *Pst* is also of economic importance (Xin and He, 2013). A major advantage of working with *Pst* in *Arabidopsis* plants is that both compatible and non-compatible interactions can be studied by using virulent and non-virulent strains, such as DC3000 and AvrRPM1, respectively. Recognition of the non-virulent protein AvrRPM1 by the disease resistance protein RPM1 from *Arabidopsis* leads to an oxidative burst with increased cytosolic calcium levels and a HR (Grant et al., 2000).

### LCBs IN PLANT IMMUNITY

**FIGURE 4:**
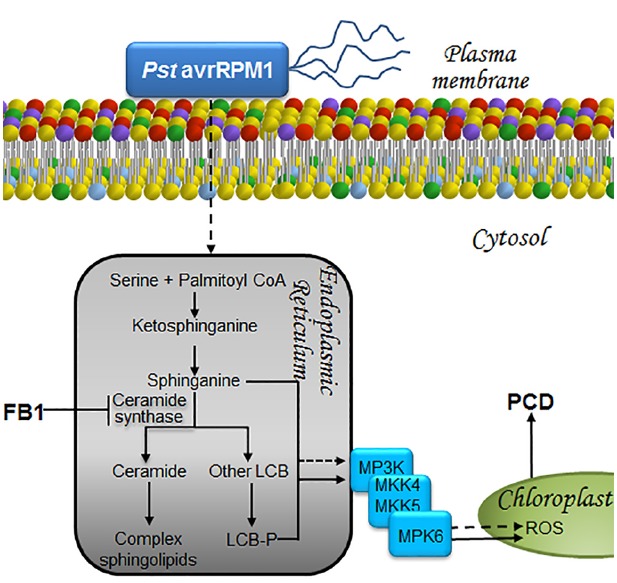
**LCBs, PA, MAPKs, and ROS in the signaling pathways that lead to cell death to prevent proliferation of biotroph pathogens.** The presence of a biotrophic or a hemibiotrophic pathogen is perceived and the information is transduced as a surge of long-chain bases at the endoplasmic reticulum in a pathway that involves MPK cascades and ROS as illustrated. As a result, cell death is unchained in a very restricted zone of the plant tissue. This avoids substrate availability for pathogen proliferation, preventing its dissemination. This programmed cell death is a fundamental part of the hypersensitive reaction or HR, a successful and effective way of defense against pathogens. Exposure to the non-virulent bacterial strain *Pst* (AvrRpm1) or to FB1, the toxin secreted by *F. verticillioides*, both result in increased levels of LCBs and LCB-Ps within the endoplasmic reticulum. Accumulation of these mediators ultimately leads to PCD through a mechanism involving the synthesis and accumulation of ROS within the chloroplast, following activation of a MAPK cascade that includes MPK6. LCB, free long-chain base; LCB-P, phosphorylated form of long-chain base; MKK4/MKK5, mitogen-activated protein kinase kinase activators of MPK6, MPK, mitogen-activated protein kinase; MP3K, mitogen-activated protein kinase kinase kinase; PCD, programmed cell death; *Pst* avrRPM1, *Pseudomonas syringae* pv *tomato* DC3000 expressing *avrRPM1*, a bacterial avirulence determinant that responds to the NDR1-dependent CC-NBS-type R gene; ROS, reactive oxygen species.

The resemblance between FB1 and LCBs makes it possible for the toxin to be recognized by the ceramide synthase of the host. As a result, FB1 inhibits the LCB acylation reaction required for ceramide formation, leading to an accumulation of LCBs. The FB1-resistant mutant *fbr11-1*, in which the *LCB1* gene is ablated, exhibits low levels of LCBs and LCB-Ps (Shi et al., 2007). LCB1 is one of the heterodimeric subunits of serine palmitoyltransferase (SPT), the enzyme responsible for the condensation of serine with palmitoyl-CoA in the first reaction of *de novo* synthesis of sphingolipids. The other subunit of SPT is LCB2a, whose genetic disruption was shown to lower the accumulation of LCBs while abolishing FB1-induced PCD (Saucedo-García et al., 2011a), indicating that LCBs lead to PCD (Figure 4). This LCB-mediated PCD response fulfills a defense mechanism to prevent the dissemination of non-compatible pathogens as *Pst* (Saucedo-García et al., 2011a). A similar result of a plant-pathogen interaction accompanied by an increase in LCB and LCB-P levels is illustrated by exposure of *Arabidopsis* to the non-virulent *Pst* strain (avrRPM1), which causes sustained elevation of endogenous LCB levels (1–24 h; Figure 4). In contrast, only a transient increase (1–5 h) is observed when the virulent *Pst* DC3000 strain is used (Peer et al., 2010).

### MAPKs AS TRANSDUCERS IN PATHWAYS LEADING TO IMMUNITY RESPONSES

MPK6 is a signaling element located downstream of LCBs whose activity is essential for the FB1-induced PCD response (Saucedo-García et al., 2011a). This link between MPK6 and PCD was recently confirmed in *Arabidopsis* and found that pretreatment with flg22 suppressed FB1-induced MPK6 phosphorylation and PCD (Igarashi et al., 2013). Since this effect occurred concurrently with reduced expression levels of the *MKK4/MKK5* genes, it suggests that their products are the activators of MPK6 (Igarashi et al., 2013). In this regard, earlier studies had demonstrated that the constitutive expression of *MKK4/MKK5* produces lesions similar to an HR (Ren et al., 2002; Figure 4).

MPK6 is another key signaling element in the non-compatible interaction with *Pst* (avrRPM1), since a mutant with a defective *mpk6* gene supports a higher level of bacteria growth compared to wild-type plants (Saucedo-García et al., 2011a). This indicates that MPK6 is essential for the control of bacterial proliferation, at least in the context of this interaction (Figure 4). Regarding MPK4, its constitutive activity is ineffective in *Pst* (avrRPM1) resistance, but is a successful negative regulator in the immune response against *Pst* (avrRPS4), suppressing the resistance response (Berriri et al., 2012).

### ROS GENERATION FROM CHLOROPLASTS AS NECESSARY TRANSDUCERS IN DEFENSE PATHWAYS

Besides the activation of the signaling pathways that induce PCD in *Arabidopsis*, FB1 causes effects at a structural level, particularly the disruption of the tonoplast (Kuroyanagi et al., 2005), chloroplast (Saucedo-García et al., 2011a), and mitochondrial membranes (Saucedo-García et al., 2011b). Tonoplast damage is noticeable, since FB1 produces an important loss of cell turgidity (Kuroyanagi et al., 2005) characterized by a distant location of organelles away from the plasma membrane (Saucedo-García et al., 2011a). In addition to tonoplast damage, FB1 activates expression of the γ-isoform of the *VACUOLAR PROCESSING ENZYME* (*γVPE*), which product shows a similar activity to caspase-1 (Kuroyanagi et al., 2005). This enzyme participates in the FB1-induced PCD response, since *Arabidopsis* mutants lacking *γVPE* expression show less intense PCD lesions compared to the wild-type plants exposed to the mycotoxin. Although the mechanism underlying the effect of γVPE on FB1-induced PCD is unknown, the MPK6-mediated signaling pathway does not appear to be involved in γVPE expression (Igarashi et al., 2013), as opposed to the heat-shock induced PCD (Li et al., 2012).

The disruption of the chloroplast membrane observed upon FB1 treatment might reflect exacerbated ROS production inside the organelle, where it accumulates (Saucedo-García et al., 2011a). This observation was recently confirmed by the detection of ROS formation in chloroplasts from FB1-treated plants (Xing et al., 2013). Likewise, *Pst* (avrRPM1) induces ROS formation in chloroplasts (Saucedo-García et al., 2011b), suggesting that both FB1 and this non-virulent strain share common signaling elements and response mechanisms, including the buildup of LCBs and LCB-Ps, MPK6 activation and ROS production in the chloroplast (Figure 4).

## TRANSDUCERS IN THE SIGNALING RESPONSE TO COLD STRESS

Plants are exposed to different types of abiotic stress with an impact on their growth and development. Sudden or unexpected decreases in temperature are one of the most important stressors, with the attendant loss of entire crops. Although many plant species are cold-sensitive, there are others that can tolerate exposure to low temperatures (Solanke and Sharma, 2008). A resistance mechanism to cold in the latter occurs through a gradual process, called acclimation, which involves the step-wise development of molecular changes during a short-time exposure to low, but not freezing temperatures (Solanke and Sharma, 2008). Cold acclimation allows plants to survive subsequent sub-zero temperatures. Exposure to temperatures between 0° and 10°C produces a chilling stress, while exposure to temperatures below 0°C causes a freezing stress (Solanke and Sharma, 2008).

### LCBs AND PA AS SIGNAL TRANSDUCERS IN RESPONSE TO COLD

Despite the limited information about the signaling pathways involved in the response to low temperature, it is known that some lipid molecules play an active role. Again, LCBs and PA appear as recurring transducers mediating responses to the same stressor.

#### Long-chain bases

**FIGURE 5:**
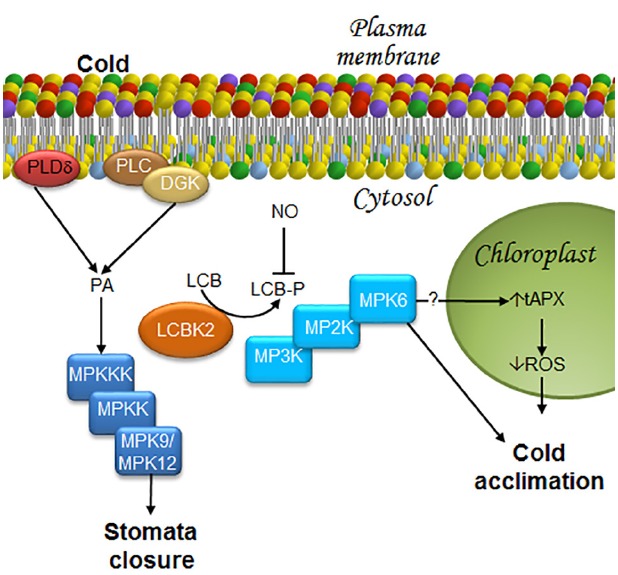
**LCBs, PA, MAPKs, and ROS in the signaling pathway in response to low temperature stress.** Stomata closure and cold acclimation are two effects caused by exposure to low temperature. In order to reach these responses, some transducers to the cold signal have been described as it is illustrated in this figure. In the case of the stomata closure, information about a decrease in water availability at the plant root due to low temperatures is sensed by the guard cells that close in order to avoid water loss. Acclimation, the other response triggered by cold exposure is very complex, since it includes metabolic and genetic reprogramming that lead to membrane remodeling and synthesis of anti-freezing or crio-protecting molecules. As a result, plants later withstand freezing temperatures. Stomata closure requires MPK9/12. Since cold stress promotes rapid production of PA through PLDδ activation, as well as the joint action of PLC and DGK, it is suggested that these enzymes function upstream of an MPK9/MPK12 cascade, as in the case of ABA-induced stomata closure. Furthermore, it has been shown that during cold acclimation, there is an increase in LCB-Ps upstream of MPK6, which can then regulate ROS accumulation mediated by tAPX in the chloroplast. Therefore, the model suggests that MPK6 could be an activator of tAPX. DGK, diacylglycerol kinase; LCB, free long-chain base; LCBK2, long-chain base kinase2; LCB-P, phosphorylated form of long-chain base; MPK, mitogen-activated protein kinase; MP2K, mitogen-activated protein kinase kinase; MP3K, mitogen-activated protein kinase kinase kinase; NO, nitric oxide; PA, phosphatidic acid; PLC, phospholipase C; PLDδ, δ isoform of phospholipase D; ROS, reactive oxygen species; tAPX, thylakoid membrane-bound ascorbate peroxidase.

During the cold-acclimation process, phosphorylated LCB levels are known to increase, particularly PHS-P (Dutilleul et al., 2012). Production of LCB-Ps is catalyzed by a LCB kinase, namely LCBK2 (At2g46090) (Dutilleul et al., 2012). Loss-of-function mutations of *LCBK2*, but not of *LCBK1* or *SPHK-1*, abolishes the ability of the plant to raise PHS-P levels during cold-acclimation (Dutilleul et al., 2012), indicating that LCBK2 is required for LCB-Ps production (Figure 5). Although PA and PHS-P share a common signaling function in ABA-mediated stomata closure, their production in response to cold stress is entirely different.

#### Phosphatidic acid

Phospholipase C and PLD are some of the first enzymes that are simultaneously activated in the first few seconds after exposing the plant to 0°C. These enzymes increase the levels of PA from hydrolysis of membrane lipids (Ruelland et al., 2002). From these two PA-generating enzymes, PLC–DGK is the major effector responsible for PA production under chilling conditions (Figure 5). Several observations support this conclusion: (a) about 20% of the PA produced under cold conditions is derived from PLD activity (Ruelland et al., 2002); (b) single- or double-KO mutants of genes associated with the cold response (*PLDα* and *PLDδ*) do not exhibit any increase in PA levels upon cold-exposure (Arisz et al., 2013); (c) an important decrease of phosphatidylinositol phosphate is observed in response to cold exposure; and (d) a rapid increase and labeling of ^32^PA occurs in cold-treated seedlings. While DGK can incorporate Pi from AT^32^P to produce ^32^P-PA, labeling of PA precursors by PLD could take longer. Such kinetics is in disagreement with the observed fast increase of ^32^P-PA (Arisz et al., 2013).

Although the participation of PLD and PLC–DGK in response to cold stress is well documented, the identity of the specific isoforms involved is unknown due to their multiplicity, as well as the complexity of the signaling networks responsible. Thus, at least 12 PLD isoforms (Qin and Wang, 2002), 9 PLC isoforms (Mueller-Roeber and Pical, 2002), and 7 DGK isoforms (Katagiri et al., 1996; Gómez-Merino et al., 2004) have been described in the *Arabidopsis* genome.

### MAPKs IN THE SIGNALING ASSOCIATED WITH THE RESPONSE TO COLD EXPOSURE

Mitogen-activated protein kinase cascades are involved in the chilling stress response in *Arabidopsis*. One of the first pieces of evidence suggesting this association was shown by Ichimura et al. (2000), who found that exposure of *Arabidopsis* to low temperatures (4°C) induced MPK4 and MPK6 activation. Twelve years later, Dutilleul et al. (2012) demonstrated that MPK6, but not MPK4, is involved in the chilling response and that MPK6 acts downstream of PHS-P accumulation (Figure 5). However, participation of other MPKs in the response to low temperatures cannot be excluded, since the *mpk9/mpk12* double-mutant exhibits partially reduced stomata closure upon cold stimulation (Jammes et al., 2009). Elsewhere, this finding suggests that activation of very similar ABA-induced mechanisms may occur in the response to low temperature, including PA, LCBs, and MPK9/MPK12 (Figure 5).

### ROS IN THE SIGNALING RESPONSE TO COLD STRESS

An oxidative burst, characterized by increased production of superoxide ion, hydrogen peroxide and free radicals is observed during cold stress. As in the case of PA and LCB-Ps, ROS involved in the *Arabidopsis* acclimation to low temperatures are produced in a cell compartment different than that involved in ABA-mediated stomata closure. While in the latter ROS are produced in the plasma membrane, they are generated in the chloroplast during acclimation. Silencing of the *THYLAKOID MEMBRANE-BOUND ASCORBATE* (*tAPX*) gene, which encodes a protein that reduces H_2_O_2_ to H_2_O and functions as a monitor of oxidative stress, is associated with oxidized chloroplast proteins, suppresses cold-responsive gene expression, and increases sensitivity to cold. These results indicate that *tAPX* silencing negatively affects cold acclimation (Maruta et al., 2012; Figure 5).

In addition to ROS, NO is a signaling molecule also involved in the acclimation to low temperatures and ABA-mediated stomata closure. However, although NO positively regulates PA synthesis during cold acclimation in other plant species (Laxalt et al., 2007; Distéfano et al., 2008), it is not involved in PLD- or PLC/DGK-dependent cold-induced PA synthesis in *Arabidopsis*. Likewise, NO is also a negative modulator of PSH-P and phosphosphingolipid synthesis (Cantrel et al., 2011). Accumulation of phosphosphingolipids is observed in the null double-mutant *nia1nia2*, which lacks expression of *NITRATE REDUCTASE* (NR), the enzyme purported to be the main source of NO generation under cold stress conditions (Cantrel et al., 2011). Moreover, *Arabidopsis* plants overexpressing the non-symbiotic hemoglobin AHb1, a protein that catabolizes NO (Perazzolli et al., 2004), exhibit reduced content of NO and increased levels of LCBs under acclimation conditions, particularly the desaturated PHS stereo isomers t18:1(8E), t18:1(8Z) (Guillas et al., 2013a; Figure 5). Although the regulation mechanism of LCB-P formation by NO is unknown, Guillas et al. (2013b) have suggested two ways: in one, NO affects the sphingosine kinase/phosphatase activities responsible of LCB-P levels, and in the other, NO participates in the availability of the sphingosine kinase substrates.

## INSIGHTS AND CONCLUDING REMARKS

The way in which different stressors engage specific and distinct transduction pathways is a very complex problem to elucidate. Signaling can be elicited by a great number of biotic and abiotic stimuli, but a general common route seems to be utilized: information must flow from the plasma membrane to the nucleus, allowing the expression of specific genes in response to the original stimulus. The resulting informational pathways are orchestrated by a large number of molecules of very diverse nature. However, the biochemical features of these transducers serve to clearly divide them into two groups: protein transducers and non-protein transducers. The former group includes receptors, G proteins, kinases, phosphatases and transcription factors. By means of their great diversity of tertiary and quaternary structures, coupled with their isospecies diversity, they all impart exquisite specificity, fine-tuning and direction to the signaling pathways in which they participate. They exhibit a high level of selectivity, both in terms of their upstream activator molecules, as well as of the molecules that they target downstream. In addition, they can be fine-tuned by regulatory effectors, and display extended half-life and specific cellular location. The non-protein transducers group is comprised by ions and small organic molecules, such as Ca^2+^, O_2_^–^, NO, H_2_O_2_, cAMP, and lipid byproducts of complex lipids. In this group, chemical diversity is more limited, with their specificity being conferred primarily by the acceptor target. Regulatory control is mainly determined by their source and extent and rate of production, their short half-life and their cellular location. These less restrictive features may have been selected to exist in multiple pathways at nodal points of control, imparting swiftness, efficiency, and the possibility of transient interaction with other signaling routes. Because of their widespread occurrence at convergent points in several signaling pathways, the role of LCBs, PA, MAPKs, and H_2_O_2_ in the response to stress has been the focus of this review. Figures 2–5 depict pathways differentially configured to contend with drought, pathogens or cold, but that however, substantiate the iterative presence of LCBs, PA, MAPKs, and H_2_O_2_ as transducers. Hereafter, they are nominated as nodes in the intracellular information wiring.

Notwithstanding the role of these transducer molecules in different signal transduction pathways, they evoke highly specific responses for a given perturbation. Much of the specificity of the response can be attributed to the site of generation of the second messenger mediators. This is particularly illustrated in the case of H_2_O_2_. As shown in Figures 2 and 3, this ROS can be produced at the plasma membrane through the activation of NADPH oxidase in response to ABA and SA, leading to stomata closure in both cases. However, H_2_O_2_ can also be produced in the apoplast through the action of peroxidase in response to chitosan-, YEL, or SA stimulation (Figure 3). Likewise, H_2_O_2_ can be generated in the chloroplast during the defense response to FB1, to infection by *Pst* (avrRPM1; Figure 4) or during chilling stress conditions (Figure 5). Therefore, the generation site of H_2_O_2_ in the different subcellular compartments seems to be crucial to elicit a unique response.

Different responses evoked by common transducers involved in diverse signaling pathways can also be explained by the existence of several possible protein effectors that may originate the same second messenger. As mentioned in the example above, H_2_O_2_ may be formed by oxidases or peroxidases in different locations. In the case of PA, for example, this can be produced by PLDα1 and PLDδ in the ABA-mediated stomata closure (Figure 2), by a PLD (of unknown identity) in response to SA (Figure 3), and by a PLDδ or PLC/DGK during cold stress (Figure 5). In a similar fashion, LCB-Ps can be produced by the tonoplast kinases SPHK1 and SPHK2 in the ABA-mediated stomata closure (Figure 2), or by LCBK2 during chilling stress (Figure 5). Importantly, a marked increase of LCB-Ps has been detected during the defense reaction elicited by FB1 in *Arabidopsis* (Figure 4; Saucedo-García et al., 2011a), although the enzyme responsible for its generation and its subcellular location remains undiscovered.

Activation of MAP kinase cascades is involved in all of the signaling pathways considered in this review. Transduction triggered by different stimuli may involve the same MPKs, as in the case of stomata closure induced by ABA, chitosan, YEL, SA, or cold exposure, wherein MPK9 and MPK12 are activated (Figures 2, 3 and 5, respectively). In contrast, activation of MPK6 is involved in flg22-mediated stomata closure, exposure to FB1 or *Pst* (avrRPM1), as well as in cold acclimation (Figures 3–5, respectively). The next logical question is how the same elements (MPK9/MPK12 or MPK6) can induce a highly specific pattern of gene expression. A possible answer may lie in the duration of MPK activation and the role of upstream immediate activators (MP2Ks). This is a topic that still remains unclear and that, together with the other queries concerning the interconnected information flow among the different transduction pathways, are still open domains to be portrayed.

## SUMMARY

The iterative presence of small molecules as LCBs, PA, and H_2_O_2_, and of some enzymes as MAPK, posts them as nodes in the intracellular information wiring due to their peculiar features as transducers.

### Conflict of Interest Statement

The authors declare that the research was conducted in the absence of any commercial or financial relationships that could be construed as a potential conflict of interest.
